# Supplementary material for: Mechanism of partial agonism in AMPA-type glutamate receptors
Source: Nat Commun. 2017 Feb 17;8:14327. doi: 10.1038/ncomms14327 (PMC5321683; doi:10.1038/ncomms14327)

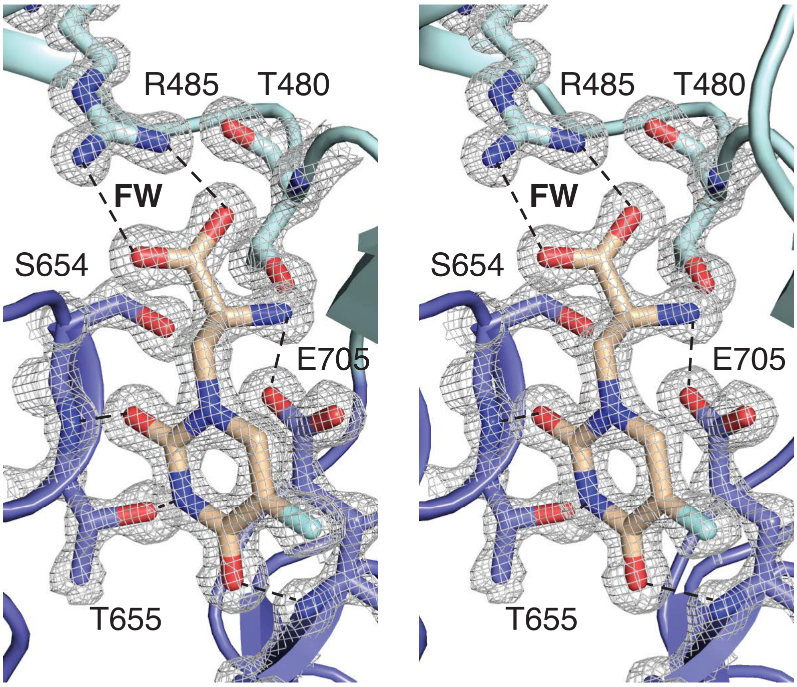

Supplementary Figure 1. Stereo view of the binding pocket showing the ligand Fluorowillardiine (FW) and surrounding residues with the 2*Fo*–*Fc* electron density map (grey mesh) contoured at 2*σ*. The upper (D1) and lower (D2) lobes of the LBD are colored pale cyan and blue, respectively. The ligand coordinating residues are labeled and shown in stick representation. Dotted lines indicate selected hydrogen bonds in the binding site.

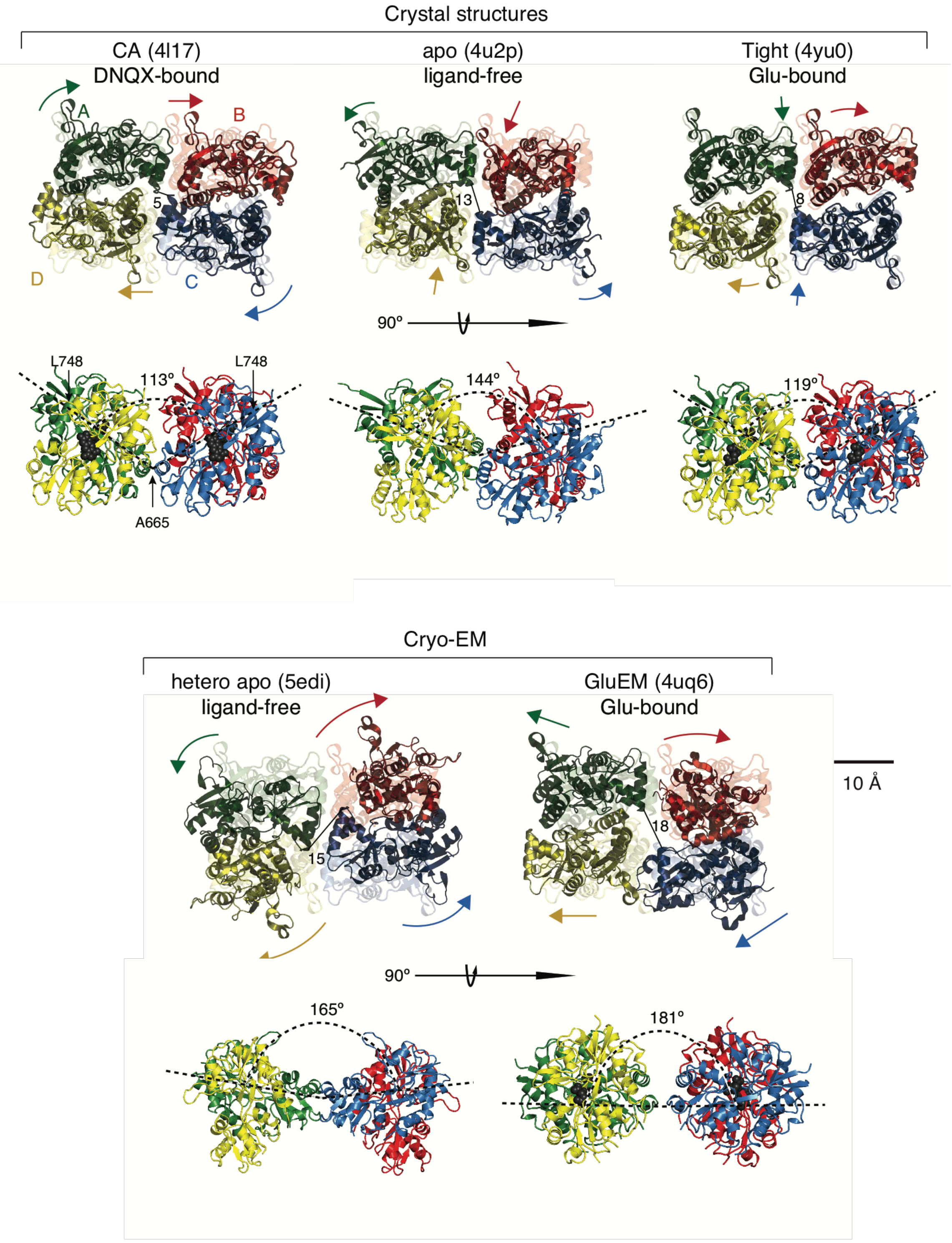

Supplementary Figure 2. Heterogeneity of LBD tetramer subunit arrangements. The indicated crystal and EM structures (viewed from above, upper rows) are superposed onto the sLBD_FW structure (water mark). Arrows illustrate the degree of movement that each of the sLBD_FW subunits

undergoes to match the aligned structure. The A-C distance in each tetramer is shown as a black line and indicated in Ångstroms. The lower rows show the same LBD structures, rotated by 90° along the x-axis to show the changes in relative dimer orientation (measured as in Figure 1, angle between vectors drawn through C-alphas of residues 665 and 748 in subunits A and C).

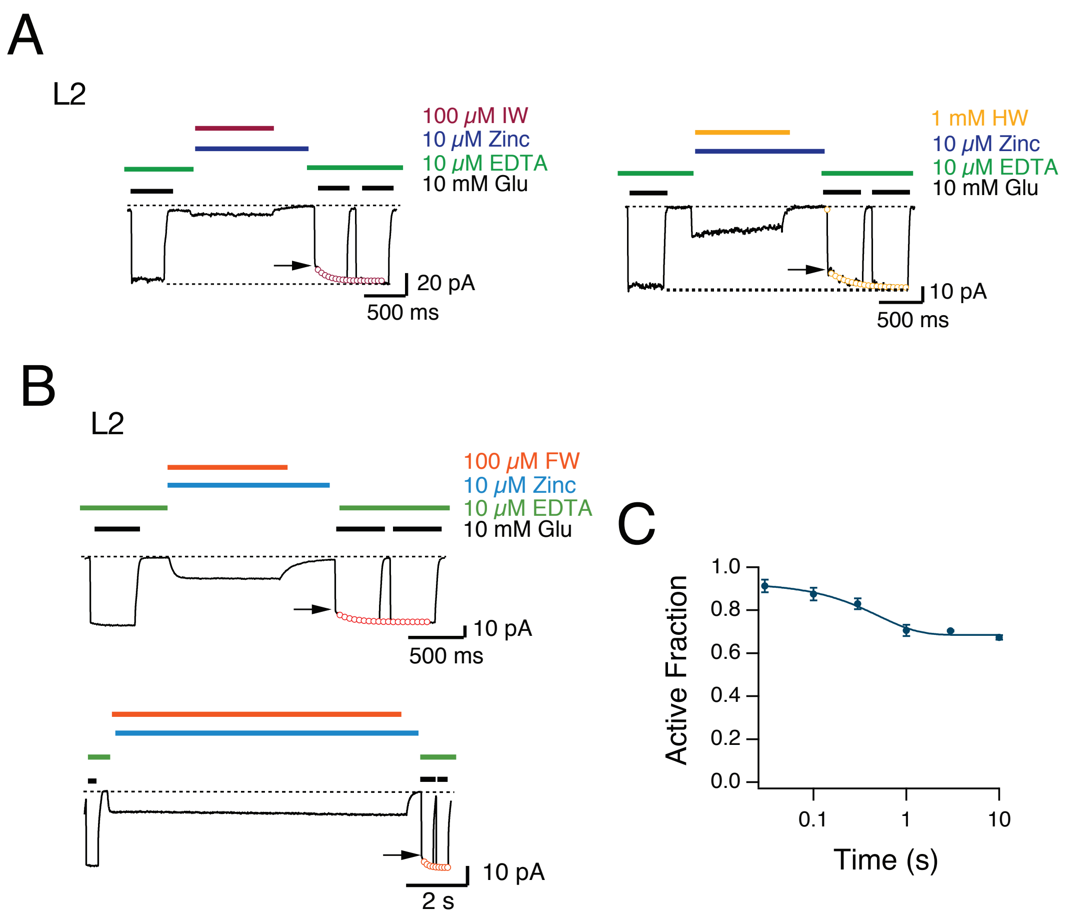

Supplementary Figure 3. Modification of the L2 mutant after the application of IW and HW and time course modification in presence of FW. (A) Patch clamp experiments showing the recovery of current in 10 mM Glutamate and EDTA following trapping in presence of Zn^{2+} 1 μM with (IW) (right panel) or (HW) (left panel). A reduction of the current after trapping was observed (indicated by arrows). Dotted lines are double exponential fit to the recovery after trapping. The time constants are summarized in the Table 1. (B) Patch clamp experiments showing the application of Zn^{2+} in presence of FW in the top panel after 1 second of application and after 10 seconds in the lower panel. A reduction of the current after trapping was observed (indicated by arrows). Dotted lines are double exponential fit to the recovery after trapping. (C) In the right panel the time application of Zn^{2+} against the active fraction is shown. All errors bars are S.E.M (n = 4).

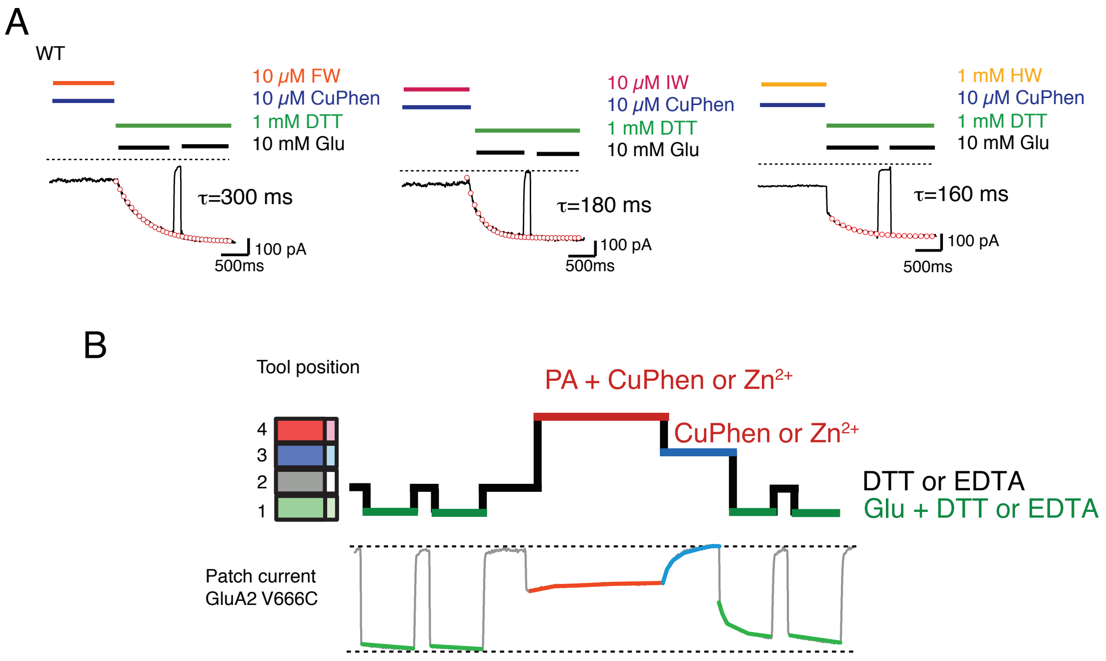

Supplementary Figure 4. Modifications to solution switches to study trapping in the presence of partial agonists. (A) Slow relaxations following a direct switch from Willardiine agonist into glutamate. The relaxations reveal, as previously reported, that the off-rate of willardiine agonists is much slower than that of glutamate (Glu τ ~1 ms[1]; τ = 300 ms, 180 ms and 160 ms for FW, IW and HW respectively). (B) In order to analyse the reduction in the active fraction due to trapping by a disulfide bond, separately from the reduction in glutamate activation due to remanant partial agoinst, we modified our application protocol to allow the partial agonist to unbind first. The top trace indicates the command position of the perfusion tool. The lower trace is the current due to the V666C mutant (gray) during the protocol. The experiment starts with the pipet positioned in front of barrel_2, that contains normal solution of Dithiothreitol (DTT) and cyclothiazide (CTZ), a drug that blocks desensitization of AMPA receptors. Subsequently, two jumps into barrel_1 containing glutamate, DTT and CTZ gives a control of the typical receptor response (green). Then, receptors were exposed to partial agonist (such as FW, IW, HW) in the presence of CuPhen (10 μM) or Zn^{2+} (10 μM) and CTZ in barrel_4 (red trace). Since partial agonists have a slow off rate it is necessary to first remove the partial agonist by jumping to barrel_3 containing a solution with CuPhen (10 μM) or Zn^{2+} (10 μM)

and (100 μM) CTZ, to hold any intersubunit bridges intact whilst partial agonist unbound whilst allowing partial agonist to unbind. Finally, by jumping back to a maximal concentration of glutamate in barrel_1, we were able to measure the active fraction of the receptor current after trapping (green trace), and the time constant of the loss of the bridge. For experiments with histidine mutants, the same protocol was used with ethylene-diamine-tetra-acetic acid (EDTA) included instead of DTT, and zinc instead of CuPhen.

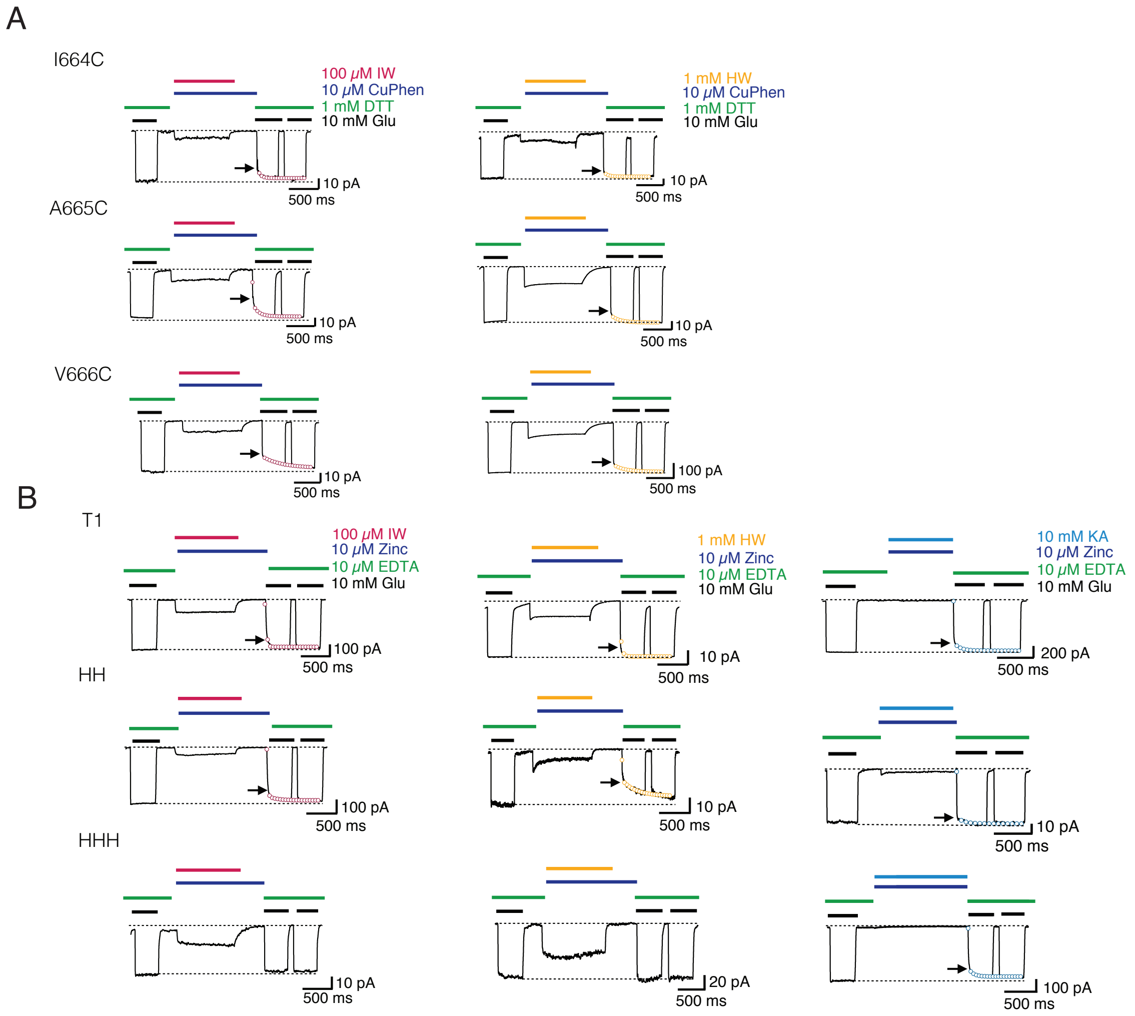

Supplementary Figure 5. Modification of the mutants of the study in the presence of IW, HW and KA. (A) Modification profile for the mutants I664C, A665C, V666C. Patch clamp experiment showing test pulses. The recovery of current in 10 mM glutamate and DTT following trapping in the presence of Zn^{2+} (1 µM) with iodowillardiine IW (left panel) or willardiine (HW) (right panel). Current reduction after modification (indicated by arrows) was observed for I664C, A665C and V666C. Dotted lines are a double exponential fit of the recovery after trapping. The time constants are summarized in Table 1. **(B)** Profile of metal bridging in IW, HW and KA. Patch clamp experiments showing the recovery of current in 10 mM glutamate and EDTA following trapping in the presence of Zn^{2+} and 1 µM of IW (right panel), HW (middle panel) and KA (left panel). A reduction of the current after trapping (indicated by arrows) was

observed for T1 and HH. The HHH mutant does not modify after the application of willardiines but a substantial reduction of the active fraction after the application of Zn^{2+} in the presence of KA was observed (arrow). Dotted lines are a double exponential fit of the recovery after trapping. The time constants are summarized in Table 1.

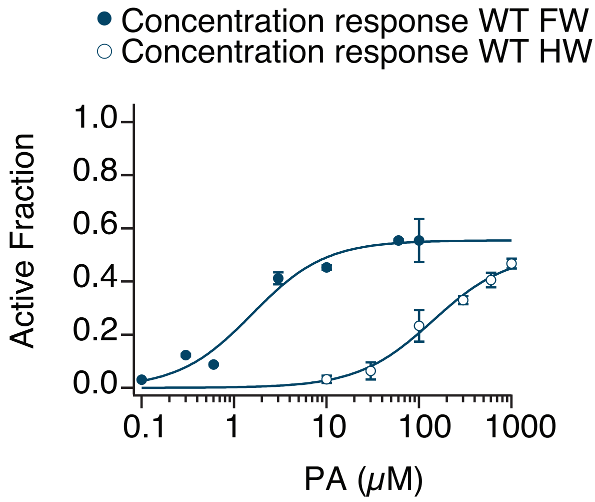

Supplementary Figure 6. Dose-response curves for WT GluA2 at different FW and HW concentrations and in the presence of 100 μM CTZ. This analysis revealed an EC_{50} of 1.5 μM for FW (closed blue circles) and an EC_{50} of 141 μM for HW (open blue circles). The maximum response (relative to glutamate) was 50 ± 9 for HW and 52 ± 3 for FW. All error bars are S.E.M. (n = 5).

Supplementary Table 1. Localization and distance between histidine residues for the four mutants T1, HH, L2 and HHH The mutated residue were modelled into the structures of the ligand free state (Apo (4u2p)) in the presence of FW (5jei), NW (4u4f), NBQX (CA (4l17)), glutamate (Tight 4yu0), KAI+RR2b+toxin (4u5d), FW+RR2b (4u1y) and FW+RR2b+tox (4u5c). The inserts show the subunits A (yellow), B (blue) and D (red). The dotted lines indicate the distance between the imidazole nitrogen atoms of the modelled histidines. Favourable rotamers were employed where possible. Distances greater than 10 Å between imidazole nitrogen are taken as too great to accommodate cross-links, even if minor conformational changes could occur.

**Mutant**	**D668H T672H K761H**	**D668H K765H D769**	**K434H H435**	**G437H K439H D456H**
Nomenclature	T1	HH	L2	HHH
Apo (4u2p)	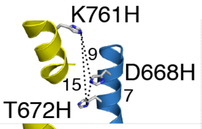	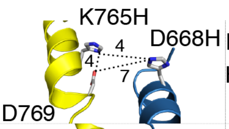	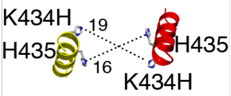	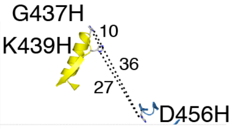
sLBD_FW (5jei)	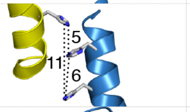	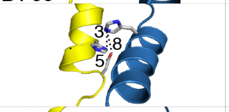	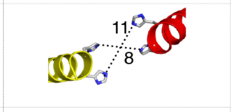	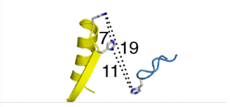
NW (4u4f)	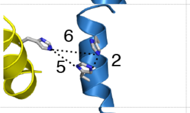	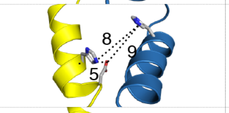	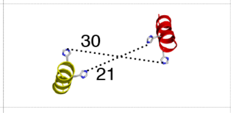	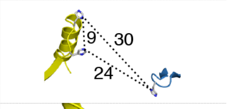
CA (4l17)	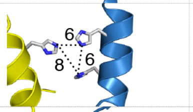	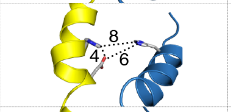	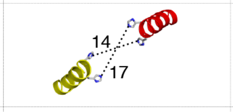	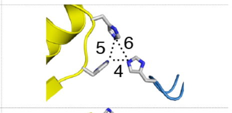
Tight (4yu0)	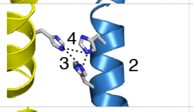	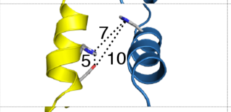	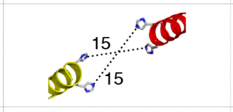	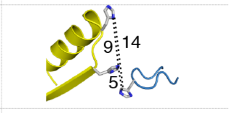
KAI (4u2q)	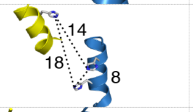	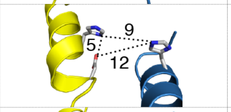	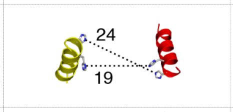	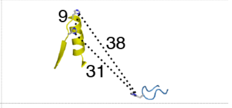
KAI+RR2b+toxin (4u5d)	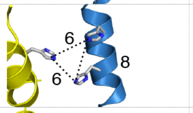	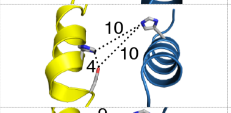	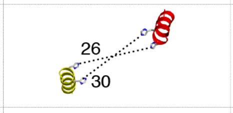	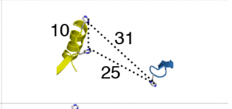
FW+RR2b (4u1y)	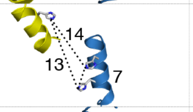	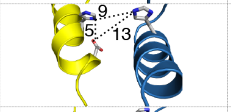	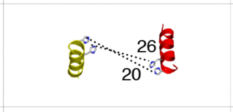	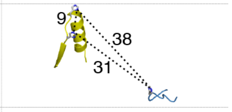
FW+RR2b+tox (4u5c)	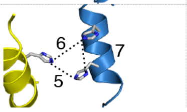	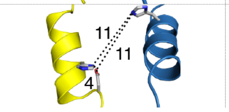	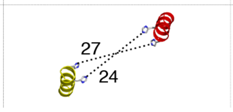	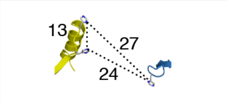

Supplementary Table 2. Agonist efficacies at the mutants used in this study. Agonist efficacies are relative to the response to 10 mM glutamate in the same patch. The bars in the bottom represent the agonist efficacies for each column (e.g. each mutant). On the right side, the efficacy of the different partial agonists is plotted for each individual mutant. All error bars are S.E.M. Each column summarises data from 5 patches.

	DTT				EDTA				
**Agonist efficacy**	**WT**	**I664C**	**A665C**	**V666C**	**WT**	**T1**	**HH**	**3 (H)**	**L2**
**FW**	58 ± 2	45 ± 4	55 ± 4	40 ± 7	58 ± 2	46 ± 1	35 ± 2	48 ± 4	52 ± 3
**IW**	31 ± 1	22 ± 2	20 ± 2	23 ± 7	31 ± 1	38 ± 1	31 ± 3	38 ± 2	37 ± 2
**HW**	52 ± 5	41 ± 2	36 ± 3	39 ± 3	49 ± 2	50 ± 5	46 ± 3	56 ± 3	50 ± 9
**KA**	12 ± 3	4 ± 2	5 ± 1	6 ± 1	12 ± 3	9 ± 4	3 ± 1	9 ± 2	6 ± 1

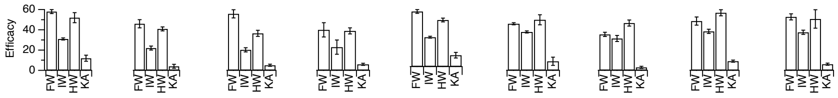

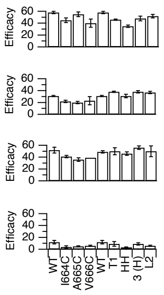

Supplement: Supplementary Information — Supplementary Figures, Supplementary Tables. [file ncomms14327-s1.pdf]
